# *Halomonas* as a chassis

**DOI:** 10.1042/EBC20200159

**Published:** 2021-07-26

**Authors:** Jian-Wen Ye, Guo-Qiang Chen

**Affiliations:** 1 School of Biology and Biological Engineering, South China University of Technology, Guangzhou 510006, China; 2 School of Life Sciences, Tsinghua University, Beijing 100084, China; 3 Center for Synthetic and Systems Biology, Tsinghua University, Beijing 100084, China; 4 MOE Key Laboratory for Industrial Biocatalysts, Dept Chemical Engineering, Tsinghua University, Beijing 100084, China; 5 Tsinghua-Peking Center for Life Sciences, Tsinghua University, Beijing 100084, China

**Keywords:** Halomonas, Metabolic engineering, Microbial chassis, Next generation industrial biotechnology, PHA, Synthetic biology

## Abstract

With the rapid development of systems and synthetic biology, the non-model bacteria, *Halomonas* spp., have been developed recently to become a cost-competitive platform for producing a variety of products including polyesters, chemicals and proteins owing to their contamination resistance and ability of high cell density growth at alkaline pH and high salt concentration. These salt-loving microbes can partially solve the challenges of current industrial biotechnology (CIB) which requires high energy-consuming sterilization to prevent contamination as CIB is based on traditional chassis, typically, *Escherichia coli, Bacillus subtilis, Pseudomonas putida* and *Corynebacterium glutamicum*. The advantages and current status of *Halomonas* spp. including their molecular biology and metabolic engineering approaches as well as their applications are reviewed here. Moreover, a systematic strain engineering streamline, including product-based host development, genetic parts mining, static and dynamic optimization of modularized pathways and bioprocess-inspired cell engineering are summarized. All of these developments result in the term called next-generation industrial biotechnology (NGIB). Increasing efforts are made to develop their versatile cell factories powered by synthetic biology to demonstrate a new biomanufacturing strategy under open and continuous processes with significant cost-reduction on process complexity, energy, substrates and fresh water consumption.

## Introduction

Due to the sustainable and environment-friendly advantages of industrial biotechnology [1–4], it has been developed for decades to produce numerous bio-based products such as bioplastics [5–7], medicines [8], chemicals, food additives [9,10] and biofuels [11–13], with an aim to gradually replace the traditional petrol-based industry. However, the long-standing challenges of contamination risk, high sterile energy input and high fresh water consumption involved in bioprocesses of current industrial biotechnology (CIB) still reduce its competitiveness in spite of the rapid development of synthetic biology [14,15].

To make industrial biotechnology competitive to chemical industry, suitable microorganisms should be constructed to solve the existing difficulties of CIB [16]. Recently, the salt-tolerating bacteria, *Halomonas* spp., are becoming the attractive candidate hosts for microbial cell factory engineering due to their strong metabolism of diverse substrates and fast growth under high salt and high pH conditions, making possible the contamination free, non-food raw materials- and seawater-consuming fermentation processes [17,18]. In contrast with the traditional model microorganisms, such as *Escherichia coli* [14,19], *Bacillus subtilis* [20], *Pseudomonas putida* [21] and others [22], the development of molecular manipulation tools and methods for *Halomonas* spp. is difficult and less effective [23]. Recent attempts have been successful to establish genetic engineering approaches [24,25] including genome editing and gene expression control [26] to construct *Halomonas* spp. into a diverse bioproduct producers.

It is important to note that, *Halomonas bluephagenesis*, one of the well-studied salt-loving wildtype microbe able to accumulate poly-3-hydroxybutyrate (PHB), has been reprogrammed to be a low-cost chassis for various biosyntheses conducted under seawater-based unsterile open fermentation [27]. This *Halomonas*-based biotechnology, namely, the ‘next-generation industrial biotechnology’ (NGIB), has exemplified successful cases in pilot-scale polyhydroxyalkanoate (PHA) productions with significant cost reduction [28], possessing promising advantages that CIB cannot have. Besides, several predominant *Halomonas* strains of industrial potential, including *Halomonas smyrnensis* AAD6 [29–31], *Halomonas* sp. KM-1 [32–34] and *Halomonas* sp. HAL1 [35], are recently developed for producing different metabolic targets, demonstrating *Halomonas* as powerful chassis in biomanufacturing based on NGIB.

The state-of-the-art developments of *Halomonas* spp. used as microbial production chassis are reviewed. Meanwhile, future perspectives and critical comments involving upstream strain engineering and downstream process optimization have been discussed in details aiming to provide constructive thoughts and possible solutions for further improvements of NGIB based on the current achievements.

## Properties of *Halomonas* spp.

*Halomonas* spp. are Gram-negative bacteria belonging to the family of Halophiles that prefer to grow in saline environments (commonly referred to NaCl or KCl), such as salt lakes and marshes, oceans or other saline areas on earth [36]. According to their preference of salt concentration under optimal growth, *Halomonas* spp. are commonly divided into two subtypes, moderate (3–15% NaCl w/v) and extreme (>20% NaCl w/v) *Halomonas* [18,36]. Most *Halomonas* spp. can survive in a wide range of temperature, reaching up to 50°C, and at alkaline conditions with a pH value over 10 [37]. Due to the fast-growth at such extreme environments, *Halomonas* spp. are grown with less contamination risks compared with non-halophilic microorganisms. *Halomonas* spp. are becoming favored hosts for developing contamination-free fermentation processes without strict sterilization [16,27].

**Figure 1:**
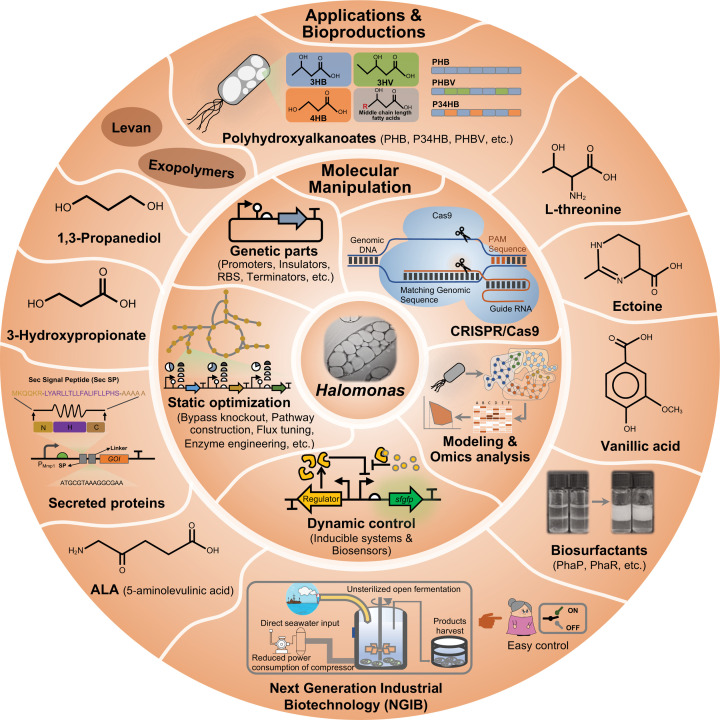
Overview of *Halomonas* engineering for biotechnological industry Many systems and synthetic biology tools and approaches, for example CRISPR/Cas9-based gene editing, omics profiling, parts mining, static and dynamic optimization methods, have been developed for *Halomonas* spp. It is thus advanced, the genetic reprogramming of *Halomonas* spp. allowing construction of high-performance *Halomonas* cell factories for production of a variety of chemicals, polyesters and proteins. A cost-effective NGIB has been developed based on extremophilic bacteria especially *Halomonas* spp. for bioproduction in various scales. Abbreviation: CRISPR, clustered regularly interspaced short palindromic repeats.

*Halomonas* are reported to possess two typical osmotic (salt-resistant) regulatory mechanisms enabling flourished growth under saline conditions: (I) accumulation of inorganic ions, such as K^+^, so as to balance the extracellular osmotic pressure of NaCl [38]; (II) production of water soluble and compatible solutes, also termed osmolytes, including ectoine, hydroxyectoine, betaine and several amino acids such as glycine, valine and proline to form an intracellular barrier resisting the influx of NaCl from saline environments [39]. For most *Halomonas* spp., mechanism-II is the preferred strategy to maintain the intra- and extra-cellular osmotic balance [40]. Interestingly, the compatible solutes ectoine and hydroxyectoine, are highly value-added compounds acting as protective agent for proteins or cells [41]; they have been commercially used in cosmetics, organ transplantation and medicinal areas. Thus, most *Halomonas* spp. are high-performing ectoine producers due to their possession of natural ectoine synthesis pathways [42–46], that can also be cloned into other hosts for enhanced ectoine production [47–49]. In addition, many *Halomonas* spp. have been reported to accumulate intracellular inclusion body, typically, PHB, one of the members of biodegradable polyesters, PHAs [3,50–52,50–52]. Moreover, increasing interests on biosurfactants, bioemulsifiers, some proteins from *Halomonas* [53–58], have turned *Halomonas* spp. into platforms for diverse bioproductions (Figure 1).

## Genetic manipulations in *Halomonas* spp.

Due to the recent growing interest in *Halomonas* spp. used as microbial chassis, many efforts have been made to develop genetic tools, such as expression vectors [59,60], promoters [24,61], ribosome-binding sites (RBSs), inducible systems [25], genome editing tools [26] and pathway tuning approaches [42,62,63], allowing genetic manipulations of *Halomonas* spp. possible for strain improvements [64]. For a given module of gene over-expression in model microbes such as *E. coli* and *P. putida*, several parts are necessary including vectors containing relative antibiotic-resistant gene(s) and replicon, promoters, RBSs, coding sequences and terminators [65]. Insulators are sometimes needed to minimize the unwanted sequence context between two different gene parts [66]. Generally, transformation of broad host range genetic parts into *Halomonas* spp. is an efficient strategy to screen suitable gene expression elements [23]. However, native plasmids isolated from Gram-negative halophiles are also good choice for cloning uses [67]. Till now, many expression vectors including pSEVA plasmids [68], shuttle vectors pWL102 and pUBP2 [69] and other broad host range or native plasmids are found to be usable in *Halomonas* spp. Accordingly, antibiotics such as chloromycetin (Cm) and spectinomycin (Spe) have been proven effective for *Halomonas* spp [70]. More importantly, a *porin* constitutive promoter library consisting of a wide range of transcriptional strengths, a novel type of T7-like inducible system and expression variances cross different expression systems were developed and characterized in *Halomonas* spp. for gene expression tuning [25,61,63] (Figure 3A). Moreover, recent efforts on whole-genome sequencing (WGS) of over 450 *Halomonas* spp. strains (Supplementary Table S1), including *H. bluephagenesis* (also termed *Halomonas* TD01), *Halomonas smyrnensis* AAD6 [71,72], *Halomonas* sp. KM-1 [73], *Halomonas* sp. HAL1 [74] and so on, provides plentiful genomic information for boosting the developments of endogenous genetic parts mining, valuable pathways identification, metabolic networks modeling [72,75] etc., which offers fundamental insights into rational microbial cell factory engineering based on *Halomonas* sp. [35,75–77].

Genome editing tools including homologous recombination and clustered regularly interspaced short palindromic repeats (CRISPR)/Cas9 have been commonly used for site-specific mutagenesis in many microorganisms [78]. However, there were less studies reported in *Halomonas* spp. [26,79] except some successful cases based on double cross-overs homologous recombination using lethal genes or helper plasmids as selection pressures. An essential gene-deficient mutant can be used as the host (in the presence of the gene encoded in a plasmid), for example, the deletion of gene *pryF* encoding orotidine-5′-phosphate decarboxylase, can significantly improve the selection pressure during mutagenesis, especially for essential gene deletion and large deoxyribonucleic acid (DNA) fragment integration [80,81]. Recently, a CRISPR/Cas9-based gene editing tool was established in *H. bluephagenesis* in the authors’ lab for engineering chromosomes, such as gene knock-down for morphology control [80], bypass deletion for product flux enhancement [82], target module integration on to the chromosome [63]. All of these efforts help turn *Halomonas* spp. into a reprogrammable chassis comparable with other model microbes (Figure 1).

Currently, conjugation is still the most commonly used genetic transformation method for *Halomonas* spp. due to the unsolved difficulty of electroporation or chemical transformation of expression vectors [79]. Therefore, many high throughput-dependent methods are not useful for *Halomonas* spp. engineering, resulting in challenges of attempts in large dataset mining and analysis when leveraging ‘Design-Build-Test-Learn’ cycle [83].

## Bioproductions by recombinant *Halomonas* spp.

**Figure 2:**
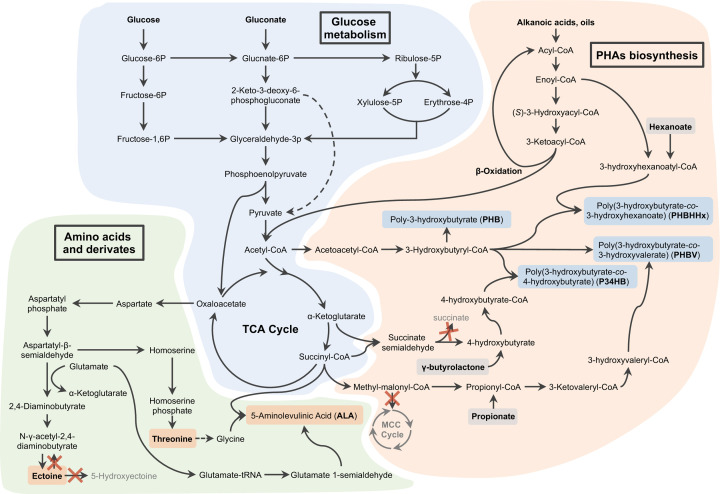
Metabolic pathways for diverse bioproductions by engineered *Halomonas* *Halomonas* spp. have been engineered to produce different chemicals and polyesters from glucose, fatty acids, gluconate or other structure-related carbon sources (light gray). Three major metabolic modules involved in glucose metabolism, PHA biosynthesis (light blue), and productions of amino acids and their derivates (light orange) are summarized according to the published studies. Crosses in red represent bypasses (letters in light gray) required to be knockout.

Due to the natural accumulation capability of PHAs and osmolytes by many isolated *Halomonas* spp., metabolic engineering on *Halomonas* spp. has attracted growing attention for enhanced production of PHA, ectoine and their derived products, levan, exopolymers and so on (Figure 1 and Table 1) [29,84]. A lot of studies have been devoted to produce diverse PHA, including PHB [27,50,85], copolyester of 3-hydroxybutyrate and 3-hydroxyvalerate (PHBV) [82], copolyester of 3-hydroxybutyrate and 4-hydroxybutyrate (P34HB) [70] from glucose, sucrose [56] and waste gluconate [28]. *Halomonas boliviensis* [46,50], *Halomonas campaniensis* [52] and *H. bluephagenesis* [28] performed well in PHA accumulation. *H. boliviensis* can utilize diverse substrates to produce high molecular weight PHB, reaching up to 1100 kilo-Daltons (kDa) [50]. *H. campaniensis*, a moderate halophile, was engineered to produce over 70 wt% PHB using kitchen waste-like mixed substrates conducted under continuous and open fermentation over a period of 65 days [52]. Notably, *H. bluephagenesis*, a predominant PHA producer isolated from Idyngo Lake, Xinjiang/China, is able to accumulate more than 90 g/l biomass containing over 80 wt% of PHB [27]. Further engineering effects have made it a diverse PHA copolymer producer, such as PHBV [82], P34HB [70] and copolyester of 3-hydroxybutyrate and 3-hydroxyhexanoate (PHBHHx) [86], when grown on glucose and/or structure-related carbon sources serving as precursors of the non-3HB monomer (Figure 2). It is important to note that the pilot-scale P34HB production by engineered *H. bluephagenesis* has demonstrated its success as an industrial chassis for NGIB [6]. In addition, recombinant *H. bluephagenesis* has displayed proven ability for productions of bio-surfactant and bio-emulsifier (PHA surface binding proteins PhaR and PhaP) [23,57], ectoine [42], l-threonine [54], 5-minolevulinic acid (ALA) [53], 3-hydroxypropoinate (3HP) [87] and many more to come, all under open unsterile conditions (Figure 2).

**Table 1:** Key achievements of bioproductions by *Halomonas*

Strains	Productions	Titer	References
*H. bluephagenesis* (*Halomonas* TD01)	Ectoine	28 g/l	[42]
	Threonine	33 g/l	[54]
	3-Hydroxypropionate	154 g/l	[87]
	5-Minolevulinic acid	0.7 g/l	[53]
*H. smyrnensis* AAD6	Levan	18.06 g/l	[31]
*Halomonas* sp. KM-1	Pyruvate	63.3 g/l	[34]

## Metabolic engineering in *Halomonas* spp.

**Figure 3:**
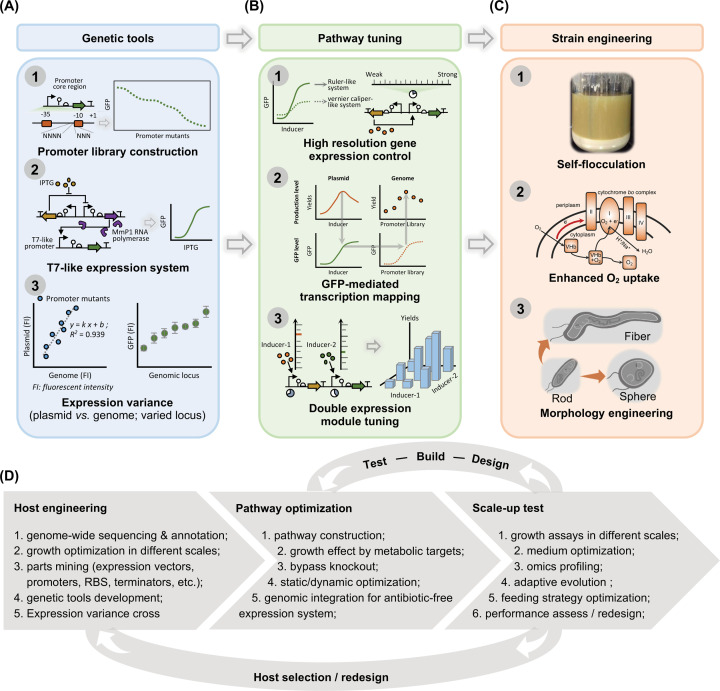
Streamlined engineering of *Halomonas* as a chassis (**A**) Genetic tools development includes promoter engineering, novel type T7-like inducible system, expression correlation cross vectors and/or different genomic locus. (**B**) Stimulus response-based pathway tuning approach enables fast and exquisite gene expression optimization on chromosome-based system in corporation of GFP-mediated transcription mapping approach. (**C**) Innovative strain engineering strategies boosts the industrial performance of engineered cells by enhancing oxygen availability, increasing the cell volume, achieving self-flocculation at the end of the growth and production processes. (**D**) Strain engineering pipeline of *Halomonas* for industrial purposes. ABbreviation: GFP, green fluorescent protein.

Metabolic engineering generally requires exquisite expression tuning of target pathways to channel the metabolic flux towards metabolic targets leveraging different static optimization approaches in addition to the commonly used methods, including enzyme engineering, promoter engineering, RBS optimization, gene over-expression (on/off-control only) and bypass knockout (Figure 2). Recently, an approach termed high-resolution gene expression control was developed for *H. bluephagenesis* based on the combination of two isopropyl-β-d-thiogalactopyranoside (IPTG)-induced systems with different dynamic ranges [63]. Similar to the length measuring tools with different scales of measuring ranges, this approach allows precise transcription tuning of target genes on chromosome directly in cross-magnitude scopes in corporation of green fluorescent protein (GFP)-mediated transcriptional mapping strategy [63]. On the basis of these successes, two orthogonal inducible systems induced by acyl homoserine lactone (AHL) and IPTG, respectively, were used to fine-tune two individual expression modules simultaneously, resulting in over 12-folds improvement of ectoine titer by engineered *H. bluephagenesis* [42]. This attempt gives a successful example for high throughput-independent strain engineering paradigm without large size library construction and labor-intensive screening process (Figure 3B), which are helpful for non-model bacterial engineering with low efficient transformation methods, such as conjugation.

In addition to the metabolic engineering approaches, several strategies have been employed to achieve strain improvements for *Halomonas* spp.: (I) morphology engineering to enlarge cell shapes for enhanced intracellular substances (such as PHA or proteins) accumulation and for benefiting sedimentation during the separation process [80]; (II) introduction of bacterial hemoglobin, Vitreoscilla hemoglobin (VHb) for enhanced oxygen uptake leading to higher cell mass and more product formation as well as aeration energy saving [88]; (III) self-flocculation for convenient separation and wastewater-less bioprocessing by recycling the supernatant of fermented broth after heat treatment and membrane processing with a recycling rate of 70–85% [89]; (IV) control of redox potential nicotinamide adenine dinucleotide plus hydrogen (NADH)/nicotinamide adenine dinucleotide plus (NAD^+^) by supplementing acetate to improve PHA and biomass accumulation (Figure 3C) [90]. All of these strategies extend ability of *Halomonas* spp.

*Halomonas* spp. have been successfully engineered in previous studies including cell engineering, pathway optimization and process scale-up (Figure 3D). Firstly, DNA sequence-based fundamental understanding of the selected *Halomonas* sp. and the genetic tools mining thereof is the cornerstone for strain engineering. Generally, the genome-wide sequencing and annotation can provide us a predictable overview of functional gene sets and metabolic networks [91]. Growth and characterization are important to obtain cultural protocols for various engineered purposes in different scales, including 96 deep well-plates, shake flasks and bench-top bioreactors, their results serve as reference-standards for growth profiling and pre-knowledge of recombinant cells without performing the scale-up studies [63], these are especially important as *Halomonas* spp. have NaCl concentrations and pH preferences.

Most importantly, optimization of genetic parts, promoter, RBS [49] and tools, promoter library construction [92], inducible systems [93], effective CRISPR-based gene editing approach [94] and alternative expression vectors, have been demonstrated to strongly improve the programmability of *Halomonas*. Screening on chromosomal locus with low expression variance can provide applicable genomic sites for efficient integrations of multiple expression modules [95]. Secondly, assembling catalytic enzymes to rewire the endogenous flux towards metabolic targets is commonly employed to achieve prototype success of target products from 0 to 1, namely pathway construction. Further optimization leveraging static regulation, mainly refers to bypass deletion and flux tuning, and dynamic regulation [96] allowing gene expression control over time and levels, is a proven strategy to generate significant breakthrough from 1 to 100. Thirdly, a rigorous scale-up test including medium and feeding strategy optimization can obtain iterative bioprocess of refinement. Accordingly, many fundamental insights can also be uncovered during the growth assays in corporation of omics profiling [97] and adaptive evolution [98], directing the redesign of cell factory by implementing ‘Design-Build-Test’ cycle, this is also true for *Halomonas* spp.

Many bioprocess-inspired strategies are useful in real cases, such as morphology control and self-flocculating cells aiming to make continuous fermentation and easier downstream separation possible. For compiling these three parts into a closed loop streamline, the interactive relationship among ‘host engineering’, ‘downstream engineering’ and ‘scale-up’ is usually missed (Figure 3D), however, they should be combined into strain engineering so that the engineering concept is used throughout the production. The NGIB concept based on extremophilic bacteria proposed by the authors’ lab has demonstrated the engineering concept up- and down-stream of the bioproduction [64]. Recent efforts have also exemplified a successful case in rebuilding the yeast system for obtaining a metabolic target-dependent (or -preferred) host to realize enhanced production of fatty acids before the execution of pathway optimization [98].

## Opportunities and challenges

Compared with the well-studied chassis especially *E. coli* and *C. glutamicum, Halomonas* spp. displaying several advantages including contamination-resistance, fast growth, seawater-based media and wide range substrates utilization, added with rich engineering tools and approaches developed recently, have become a promising platform for NGIB. A variety of products have been successfully produced with some of them scaled up to at least 5000 liters bioreactors under open unsterile conditions. Since *Halomonas* spp. can be engineered as convenient as *E. coli*, their potentials will be at least as promising as *E. coli* which has been employed to produce many bio-based products in various scales. *Halomonas* spp.-based NGIB has provided a versatile low-cost platform of biomanufacturing to meet the increasing demand of sustainable development that chemical industry and CIB cannot realize.

More efforts and attempts should be made to strengthen the sciences and technological sites of NGIB for overcoming the accompanying challenges. For example, the development of high throughput genetic transformation methods to enable generation of large size datasets from clones [49], establishments of multigene pathway tuning strategy [93] and dynamic control systems [99], exploration of high cell density cultivation and induction technology [100], enhancement of substrates to product conversion efficiency, controllable cell morphology changes for better growth, production and downstream processing. The coming joint efforts offer more possibilities for *Halomonas* as a chassis comparable with *E. coli* yet with more advantages.

## Summary

- *Halomonas* spp. as chassis are able to grow rapidly in saline and alkaline environments allowing contamination-free cultivation under open conditions.
- *Halomonas* spp. display advantages including fast growth, seawater-based medium and a wide substrate range as well as simple cultivation processes.
- Similar to *E. coli, Halomonas* spp. can be engineered to produce diverse products including several PHAs, chemicals, food additives and proteins.
- *H. bluephagenesis* is one of the most promising hosts as it allows convenient genetic manipulations and process scale-up under open unsterile conditions.
- NGIB based on *Halomonas* spp. as chassis provides a competitive solution for overcoming the challenges of high energy and fresh water consumption based on CIB.

## Supplementary Material

Supplementary Table S1

## Abbreviations

AHL: acyl homoserine lactone
CIB: current industrial biotechnology
CRISPR: clustered regularly interspaced short palindromic repeats
DNA: deoxyribonucleic acid
IPTG: isopropyl-β-d-thiogalactopyranoside
NGIB: next-generation industrial biotechnology
P34HB: poly(3-hydroxybutyrate-*co*-4-hydroxybutyrate)
PHA: polyhydroxyalkanoate
PHB: poly-3-hydroxybutyrate)
PHBV: poly(3-hydroxybutyrate-*co*-3-hydroxyvalerate)
RBS: ribosome-binding site
